# Host genetic background rather than diet-induced gut microbiota shifts of sympatric black-necked crane, common crane and bar-headed goose

**DOI:** 10.3389/fmicb.2023.1270716

**Published:** 2023-10-12

**Authors:** Yeying Wang, Zhengmin Long, Yu Zhang, Xianyu Li, Xu Zhang, Haijun Su

**Affiliations:** ^1^Key Laboratory of State Forestry Administration on Biodiversity Conservation in Karst Mountainous Area of Southwestern of China, School of Life Sciences, Guizhou Normal University, Guiyang, Guizhou, China; ^2^Research Center for Biodiversity and Natural Conservation, Guizhou University, Guiyang, Guizhou, China; ^3^Guizhou Caohai Observation and Research Station for Wet Ecosystem, National Forestry and Grassland Administration, Bijie, Guizhou, China; ^4^College of Forestry, Guizhou University, Guiyang, Guizhou, China

**Keywords:** black-necked crane, common crane, bar-headed goose, gut microbiota, diet

## Abstract

**Introduction:**

Gut microbiota of wild birds are affected by many factors, and host genetic background and diet are considered to be two important factors affecting their structure and function.

**Methods:**

In order to clarify how these two factors influence the gut microbiota, this study selected the sympatric and closely related and similar-sized Black-necked Crane (*Grus nigricollis*) and Common Crane (*Grus grus*), as well as the distantly related and significantly different-sized Bar-headed Goose (*Anser indicus*). The fecal samples identified using sanger sequencing as the above three bird species were subjected to high-throughput sequencing of *rbcL* gene and *16S rRNA* gene to identify the feeding types phytophagous food and gut microbiota.

**Results:**

The results showed significant differences in food diversity between black-necked cranes and Common Cranes, but no significant differences in gut microbiota, Potatoes accounted for approximately 50% of their diets. Bar-headed Geese mainly feed on medicinal plants such as *Angelica sinensis, Alternanthera philoxeroides*, and *Ranunculus repens*. Black-necked cranes and Common Cranes, which have a high-starch diet, have a similar degree of enrichment in metabolism and synthesis functions, which is significantly different from Bar-headed Geese with a high-fiber diet. The differences in metabolic pathways among the three bird species are driven by food. The feeding of medicinal plants promotes the health of Bar-headed Geese, indicating that food influences the functional pathways of gut microbiota. Spearman analysis showed that there were few gut microbiota related to food, but almost all metabolic pathways were related to food.

**Conclusion:**

The host genetic background is the dominant factor determining the composition of the microbiota. Monitoring the changes in gut microbiota and feeding types of wild birds through bird feces is of great reference value for the conservation of other endangered species.

## 1. Introduction

The gastrointestinal tract of vertebrates is considered a huge “reservoir” that harbors various microbial communities (Anthony et al., 2021). The host provides a suitable environment and nutrients for the microbes, while the microbes assist in digestion and nutrient acquisition (Kohl and Dearing, 2016), prevent feather degradation (Kohl et al., 2017), and regulate immunity against pathogens (Kreisinger et al., 2015). Most of the microorganisms in the gut provide additional beneficial metabolic pathways to the host, which helps improve the host’s physiology and health (Cisek and Binek, 2014). The composition of the gut microbiota is influenced by genetic factors (Clarke et al., 2012), environmental factors (Burns et al., 2016), and diet (Rosenberg and Zilber-Rosenberg, 2018). Dysbiosis of the gut microbiota can lead to rumen acidosis (Jami et al., 2013), obesity (Ley et al., 2005), diabetes (Pascale et al., 2019), cancer (Raza et al., 2019), intestinal diseases such as irritable bowel syndrome (Bhattarai et al., 2017), and reduced gut microbiota diversity is generally regarded as a hallmark of gut microbiota imbalance (Valdes et al., 2018). Research has shown that the gut microbiota in animals is dynamic and its abundance changes with environmental factors, thus adapting to the environment (Zhao et al., 2022). The host of the gut microbiota acts like an ecosystem engineer, always knowing how to manipulate the microbial general system-level properties by regulating the abundance changes of the gut microbiota through a series of positive and negative feedbacks, in order to make the host better adapted to the environment (Coyte et al., 2015). The factors that influence the structure of the gut microbiota mainly include environmental factors, dietary differences, behavioral habits, and host genetic background factors (Perofsky et al., 2019), among which dietary differences and environmental factors have a greater impact on the structure of the gut microbiota than other factors (Muegge et al., 2011; Delsuc et al., 2014; Sanders et al., 2015), especially the host’s diet, which can not only influence but also determine to some extent the composition of the gut microbiota (Kohl et al., 2014). For example, studies on wintering hooded cranes (*Grus monacha*) and capercaillie (*Tetrao urogallus*) in Shengjin Lake have shown that the composition of gut microbiota changes with the variation of food resources (Zhao et al., 2017), and studies on geese also show that dietary differences influence the composition of gut microbiota (Yang et al., 2016). The above studies are conducted on single species, and the influence of environmental factors on species’ gut microbiota cannot be ruled out. Therefore, comparative studies of gut microbiota in sympatric symbiotic species, which exclude the influence of environmental factors, can more accurately reveal the factors that influence gut microbiota.

Different species coexisting in the same habitat is a fascinating phenomenon. Exploring the mechanisms of coexistence of different species in the same location at a microscopic level helps us better understand the relationship between host and gut microbiota, and also allows us to understand the dietary preferences of different species under similar dietary conditions (Fu et al., 2021). Previous studies have shown that three species that depend on farmland in the same region, *Otis tarda dybowskii*, *Grus grus*, and *Fulica atra*, maintain distinct gut microbiota structures during the wintering period in the same habitat, despite similar external factors (Lu et al., 2022). Researchers believe that species symbiosis is promoted by the gut microbiota, which is independently driven by each host (Lu et al., 2022). In a study on the co-distribution of Great Bustards and Common Cranes, differences in gut microbiota abundance and diversity were found between the two species. Additionally, the gut microbiota of the same hosts at different wintering sites also showed differences, indicating that hosts and diets collectively induce changes in the gut microbiota. The researchers found that hosts drive the structure and function of the gut microbiota, while food drives the differentiation of metabolic pathways in the gut microbiota (Li et al., 2021). On the contrary, the co-distribution of yak and plateau pika, which coexist in the same habitat, promotes the diversity and similarity of microbiota through mutual utilization of gut microbiota (Fu et al., 2021), which is completely opposite to the results of the study on Great Bustards, Common Cranes, and Common Coot. These findings are also slightly different from previous studies on individual species.

In the Caohai National Nature Reserve in Guizhou Province, China, the Black-necked Crane (*Grus nigricollis*), Common Crane (*Grus grus*), and Bar-headed Goose (*Anser indicus*) are coexisting bird species that share foraging grounds. The Black-necked Crane, belonging to the order Gruiformes, family Gruidae, and genus Grus, is a large wading bird species. It is one of the few species of cranes that inhabit wetlands in the Qinghai-Tibet Plateau and Yunnan-Guizhou Plateau at altitudes of 2500–5000 m. This species is listed as “Near Threatened” on the International Union for Conservation of Nature (IUCN) Red List. After analyzing the gut microbiota structure of the Black-necked Crane in six wintering sites in China, it was speculated that the core and unique microbial communities of the Black-necked Crane at different wintering sites might be caused by differences in dietary structure (Wang et al., 2020). The Common Crane belonging to the order Gruiformes, family Gruidae, and genus *Grus*, is classified as “Least Concern” on the IUCN Red List. By comparing and analyzing the microbiota in fecal samples collected from unharmed Common Cranes, it was found that non-invasive sampling better represented the fecal microbiota of the hosts compared to capture-based sampling (Turjeman et al., 2023). The dietary research on Common Cranes is currently lacking. However, observations have shown that they primarily consume plant-based food during the wintering period. The Bar-headed Goose belongs to the order Anseriformes, family Anatidae, and genus *Anser*. The phylum Firmicutes dominates the gut microbiota of Bar-headed Geese, followed by the phyla Bacteroidetes, Actinobacteria, and Proteobacteria. In captivity, Bar-headed Geese have lower reproductive rates compared to their wild counterparts, possibly due to the lack of certain foods (Wang et al., 2017). Bar-headed Geese primarily feed on plant-based foods such as leaves, stems, grasses of the Poaceae and Cyperaceae families, as well as seeds from Leguminosae plants. They also consume mollusks, mollusks, and other small invertebrates (Wang et al., 2016a,b). However, during the stable wintering period in Caohai wetland, where the weather is cold and animal-based food is scarce, all three bird species primarily consume plant-based food.

To reveal the foraging strategies and gut microbiota composition of the coexisting species under the same environmental conditions, this study selected the Common Crane and Black-necked Crane, which have similar body sizes and exhibit evident food competition, as well as the Bar-headed Goose, which has a smaller body size and no food competition advantage. Non-invasive sampling methods were used to collect feces from these bird species in the same foraging ground. Universal primers for geese and cranes were used to identify the fecal sources, and high-throughput sequencing of the plant chloroplast *rbcL* gene was conducted to determine the plant-based food composition of the three species and clarify their coexisting foraging strategies. High-throughput sequencing of the bacterial 16S rRNA V3-V4 region was performed to reveal the gut bacterial composition of the three species. Multiple analysis methods were employed to establish the relationship between food and gut bacteria and clarify the influence of food and hosts on the gut microbiota of the coexisting bird species.

## 2. Materials and methods

### 2.1. Sample collection

**FIGURE 1:**
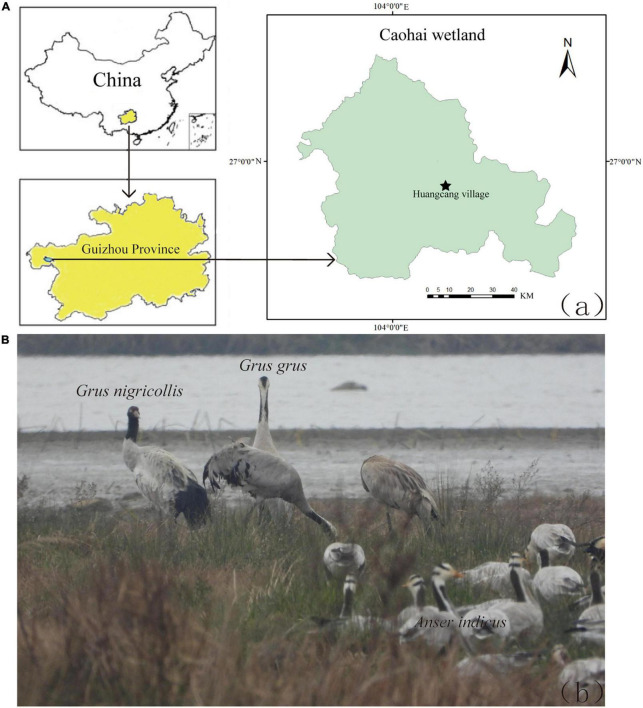
**(A)** Sampling sites of Black-necked Crane, Common Crane and Bar-headed Goose in Huangcang Village, Caohai wetland, Guizhou province. **(B)** Black-necked Cranes, Common Cranes and Bar-headed Geese overwinter together in Caohai wetland.

From January 2nd to January 7th, 2020, 112 fecal samples were collected from Black-necked Cranes, Common Cranes, and Bar-headed Geese distributed in Huangcang Village, Caohai Wetland (104°13′5.588″; 26°54′13.604″) (Figure 1). When collecting fecal samples, patience was required to wait near the foraging grounds until the bird flocks left before sampling. To minimize contamination, sterile tweezers were used to collect feces that were more than 5 meters away from the soil, and the collected feces were placed in a portable icebox. Subsequently, they were transported to the laboratory in a vehicle refrigerator and stored in a −20°C freezer for subsequent fecal DNA extraction.

### 2.2. Total DNA extraction, PCR amplification, and sequencing

Total genomic DNA was extracted from a 0.5 g portion of each fecal sample using the Stool Genomic DNA Kit (Beijing ComWin Biotech Co., Ltd., Beijing, China) following the manufacturer’s protocol. The concentration and purity were detected by quantitative fluorescence analysis (BioTek, Winooski, VT, USA). For we were not sure whether these feces were fresh and which was from the Black-necked Crane, Common Crane and Bar-headed Goose, so we used the specific primers BZ-For (5′-TCAGGGCCATACCTTGGTT-3′)/BZ-Rer (5′-TTCAGTGCCGTGCTTTGTTT-3′) of cranes D-loop genes (Sorenson et al., 1999) and primers L14990(5′-AACATCTCCGCATGATGAAA-3′) (Kocher et al., 1989)/H15298 (5′-CCCTCAGAATGATATTTGTCCTCA-3′) (Johnson and Sorenson, 1998) to amplificate the D-loop region and cytb of extracted DNA separately by the PCR Amplifier (GeneAmp 9700, ABI, USA). The PCR products with target band were send to Sangonbio (Sangon Biotech Co., Ltd., Shanghai, China) to perform Sanger sequencing. The sequencing results were aligned with the NCBI Blast database. To avoid fecal samples coming from the same individual, only one identical sequence was retained. Additionally, to eliminate bias caused by differences in sample size, a final confirmation was made using 9 fecal samples from Black-necked Cranes, labeled as GN1-GN9 with the group name GN, 9 fecal samples from Common Cranes, labeled as GG1-GG9 with the group name GG, and 9 fecal samples from Bar-headed Geese, labeled as AID1-AID9 with the group name AI. The food composition of Black-necked Crane, Common Crane and Bar-headed Goose was detected by primers 3-F (5′-ATGTCACCACCAACAGAGACTAAAGC-3′) and 3-R (5′-CGTCCTTTGTAACGATCAAG-3′) (Poinar et al., 1998; Hofreiter et al., 2000) for the chloroplast gene *rbcL*, and the gut microbiota was detected using universal primers 338 F (5′-ACTCCTACGGGAGGCAGCAG-3) and 806 R (5′-GGACTACHVGGGTWTCTAAT-3′) for bacterial 16S rDNA V3–V4 region (Mori et al., 2014). The PCR amplification system and reaction conditions all according to the previous reports (Wang et al., 2022). After recycling the target strip using the QIAquick Gel Extraction Kit (Qiagen, Hilden, Germany), the products were sent to the Personalbio (Shanghai Personal Biotechnology Co., Ltd., Shanghai, China) for high-throughput sequencing using the Illumina MiSeq system (Illumina, San Diego, CA, USA) according to the manufacturer’s instructions.

### 2.3. Data analysis

The standard operating procedure with QIIME2 (Bolyen et al., 2019) was used to identify and eliminate interrogative sequences. The DADA2 method has not yet been adapted to amplicon *rbcL*, it was used for primer removal, quality filtering, and denoising of 16S rRNA V3–V4 gene (Callahan et al., 2016), then we got a feature table (raw ASV table) and representative ASV sequences. The 16S *rRNA* gene of bacteria was evaluated by using the Greengenes Database (DeSantis et al., 2006). For *rbcL*, Vsearch was used for splicing and chimera detection (Rognes et al., 2016). The remaining high-quality sequences were clustered into operational taxonomic units (OTUs) at a 97% sequence identity threshold (Rognes et al., 2016). OTUs accounting for <0.001% of total sequences across all samples were discarded. The sequences of the *rbcL* were compared by searches against the NCBI blast in the nt database (Nilsson et al., 2006). The representative ASV or OTU sequences that failed to be assigned to known taxa were identified as “unclassified.”

Sequences were randomly sampled to draw the rarefaction curve to examine the sequencing effectiveness using QIIME2 (Bolyen et al., 2019). According to the taxonomic annotation and selected samples, the plant-based components of the diet at the family level and species level were evaluated, and the compositions of the gut microbiota at the phylum, genus and species levels were determined. Alpha diversity indices including Chao index (Chao, 1984), Shannon index (Shannon, 1948), Simpson index (Simpson, 1949), and observed species were calculated to evaluate the richness and diversity of plant-based dietary components and the gut microbiota composition using QIIME2 (Bolyen et al., 2019), and the data were visualized using box charts to represent differences in alpha diversity, the differences was tested using the Wilcoxon rank sum test among groups GN, GG and AI using R (v4.1.3; R Core Team, 2020). To measure inter-sample diversity, beta diversity analysis and analysis of similarities (ANOSIM) among groups as well as a principle coordinates analysis (PCoA) (Oksanen et al., 2010) based on Bray–Curtis distances (Bray and Curtis, 1957) (to obtain corrected *P*-values and 95% confidence intervals) were performed using R package (v4.1.3; R Core Team, 2020).

The subsequent analysis was performed following the guide on the Genescloud Platform of Personalbio, a free online platform for data analysis.^1^ To identify the inherent patterns of co-occurrence or co-exclusion of specific microbial communities in the three bird species under the influence of temporal and spatial changes and environmental processes, we used the abundance data of all ASVs in the samples. ASVs with a total sequence count less than 10 were filtered out. We used the SparCC algorithm to construct correlation matrices for the gut bacteria of the three bird species. The filtering threshold for correlation values was determined using the random matrix theory. The induced_subgraph function in the igraph package was used to extract the dominant bacterial subnetworks based on the abundance of ASV nodes, selecting the top 100 nodes with the highest average abundance, and then visualizing the subnetworks using the ggraph package. Next, we used the Bray-Curtis distance algorithm to construct clustering trees. The clustering method was set to average, and the clustering tree was combined with bar plots to simultaneously illustrate the similarity of plant-based food at the inter-group and intra-group family levels, as well as the similarity of gut microbiota at the inter-group and intra-group genus levels. After exploring the differences and similarities in microbial community composition, we used the LEfSe (LDA Effect Size) analysis to identify stable differentiating species in plant-based food and gut bacteria between groups. We used the one-against-all (less strict) comparison strategy and performed Wilcoxon tests to determine the significance of inter-group differences. The LDA threshold was set as 3 in plant-based components and 4 in gut microbiota. Spearman correlation coefficients were obtained to quantify relationships between the gut microbiota and plant-based food components (with a relative abundance of more than 1% in the tree birds as a threshold).

The metabolic functions, disease-related functions, generation of precursor metabolite and energy and biosynthesis functions of bacterial communities were predicted using the KEGG database,^2^ EggNOG database, and MetaCyc database^3^ using PICRUSt (Langille et al., 2013). The Wilcoxon rank-sum test was used to analyze the differences.

## 3. Results

### 3.1. Sequencing analysis

Illumina MiSeq sequencing of *rbcL* yielded 2,829,890 effective data (Supplementary Table 1) and sequencing of the V3-V4 region of bacterial 16S *rDNA* yielded 2,056,926 effective data (Supplementary Table 2). The average length of the rbcL sequence was 202 bp, and the average length of the 16S rDNA sequence was 430 bp. According to the rarefaction curves of shannon (*rbcL* and *16S rRNA*), the sequncing depth of each sample reached saturation, which means the microbial and diet communities of all the samples were well represented (Supplementary Figure 1). For gut microbiota, the Black-necked Crane had 4 phyla, 7 classes, 13 orders, 25 families, 34 genera and 16 species; the Common Crane had 4 phyla, 7 classes, 13 orders, 24 families, 34 genera and 17 species; and the Bar-headed Goose had 3 phyla, 7 classes, 13 orders, 24 families, 38 genera and 15 species. For plant-based components, the Black-necked Crane had 1 phyla, 11 classes, 14 orders, 23 families, 35 genera and 17 species; the Common Crane had 1 phyla, 12 classes, 15 orders, 22 families, 33 genera and 17 species; and the Bar-headed Goose had 1 phyla, 9 classes, 11 orders, 19 families, 38 genera and 21 species. Based on the above data, it can be seen that the identification rate of genus level is higher than that of other levels, so the subsequent analysis chooses level genus or above.

### 3.2. Analysis of gut microbiota composition and diversity in Black-necked Cranes, Common Cranes, and Bar-headed Geese

**FIGURE 2:**
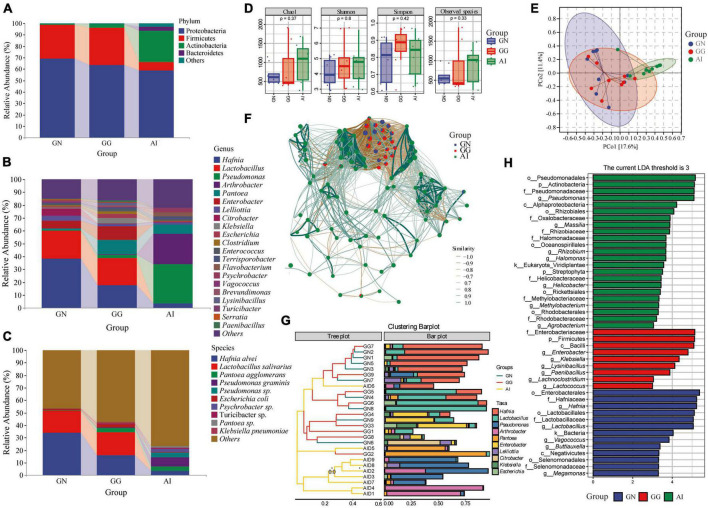
The composition of gut microbiota with relative abundance greater than 1% at the phylum **(A)**, genus **(B)**, and species **(C)** levels. Alpha diversity in gut microbiota **(D)** of Black-necked Cranes, Common Cranes and Bar-headed Geese based on Chao1, Shannon, Simpson and Observed species indexes. PCoA analysis **(E)**, Correlation network analysis **(F)**, Cluster analysis **(G)** and LEfSe analysis **(H)** of gut microbiota in Black-necked Crane, Common Crane, and Bar-headed Goose.

The *16S rRNA* gene was used to determine the gut microbiota composition of Black-necked Crane, Common Crane and Bar-headed Goose. There were four phylum with an average relative abundance greater than 1, 69.26, 63.61, and 58.83% Proteobacteria, 29.51, 32.40, and 7.43% Firmicutes were in group GN, GG and AI respectively. 2.99 and 27.13% Actinobacteria were detected in group GG and AI. Bacteroidetes (3.42%) was only found in group AI. At the genus level, 9 genus detected in group GN had relative abundances of more than 1%, including *Hafnia* (41.41%), *Lactobacillus* (21.75%), *Pantoea* (1.16%), *Enterobacter* (6.09%), *Lelliottia* (3.77%), *Citrobacter* (5.49%), *Escherichia* (1.04%), *Enterococcus* (1.26%) and *Vagococcus* (1.48%). In group GG, 14 genus had a relative abundance of more than 1%, including *Hafnia* (17.62%), *Lactobacillus* (21.12%), *Pseudomonas* (2.49%), *Pantoea* (11.13%), *Enterobacter* (10.69%), *Lelliottia* (1.53%), *Klebsiella* (4.34%), *Escherichia* (2.74%), *Clostridium* (2.34%), *Enterococcus* (1.43%), *Terrisporobacter* (1.38%), *Brevundimonas* (1.42%), *Lysinibacillus* (1.72%), and *Paenibacillus* (1.21%) (Figure 2A). 10 of the top 21 genus in group AI had a relative abundance of more than 1%, including *Hafnia* (3.47%), *Pseudomonas* (30.04%), *Arthrobacter* (23.59%), *Pantoea* (7.31%), *Lelliottia* (1.68%), *Enterococcus* (1.04%), *Terrisporobacter* (2.19%), *Flavobacterium* (3.13%) and *Psychrobacter* (2.86%) (Figure 2B). At the species level, although the detection rate is low, we can still find some bacteria with relative abundance greater than 1%, *Hafinia alvei* was detected with the relative abundance 34.10, 16.05, and 3.45% seperately in GN, GG and AI. *Lactobacillus salivarius* was detected in GN and GG with the relative abundance 17.59 and 18.22% seperately. *Pantoea agglomerans* was detected in GG and AI *with* the relative abundance 3.83 and 3.48% seperately. *Pseudomonas graminis* (7.34%), *Pseudomonas sp.*(3.98%), *Psychrobacter sp.*(2.74%), *Lactobacillus salivarius* (17.59%), *Angelica sinensis* (33.15%), *Alternanthera philoxeroides* (13.26%) and *Pantoea sp.* (1.40%) were only detected in group AI. *Klebsiella pneumoniae* (1.31%) was only detected in group GG (Figure 2C).

Alpha diversity measures were calculated for gut microbiota for a comparison among the three groups (Figure 2D). Values for the chao1 and observed species indexes were higher and the other two indexes were lower in group GN than in group GG, there were all no significant difference. Values for all the four indexes were higher in group AI than GN, but there were still no significant difference. Values for chao1, shannon and observed species index were lower in group GG than in group AI, apart from the simpson index, they were all with no significant difference.

To further analyze the gut microbiota differences among groups, we used PCoA and ANOSIM (Figure 2E and Table 1). ANOSIM indicated that the difference between group GN and AI, GG and AI showed *R* > 0 (Table 1), which means the between-group difference was greater than the within-group difference. However, the difference between group GN and GG showed *R* = −0.027263 < 0, which means the between-group difference was smaller than the within-group difference. The differences between group GN and GG had no significant differences (*P* = 0.636 > 0.05), while the differences between GN and AI, GG and AI were all showed significant differences (*P* = 0.01, Table 1).

**TABLE 1:** The ANOSIM analysis of ASVs based on Bray_Curtis between groups.

Group 1	Group 2	Sample size	ANOSIM statistic R	*p*-value
All	–	27	0.449931	0.001
GN	GG	18	−0.027263	0.636
GN	AI	18	0.729595	0.001
GG	AI	18	0.680384	0.001

To explore the similarities and differences in specific microbial communities of three species in response to temporal and environmental changes, we constructed an association network of the gut microbiota of Black-necked Cranes, Common Cranes, and Bar-headed Geese (Figure 2F). We found that Black-necked Cranes and Common Cranes shared many modules, while there were almost no shared modules between these two species and Bar-headed Geese. This further suggests that, compared to Bar-headed Geese, Black-necked Cranes and Common Cranes have more similar gut microbiota under the influence of temporal and environmental changes.

To identify the composition of gut microbiota that was similar or dissimilar between and within groups, we conducted cluster analysis and presented it using an interactive bar plot (Figure 2G). We found that samples from the GN (Gut microbiota of Black-necked Cranes) and GG (Gut microbiota of Common Cranes) groups exhibited higher similarity and were mostly clustered together, while except for samples AID6 and AID5, all 7 samples from the AI (Gut microbiota of Bar-headed Geese) group were clustered together. Among the top 10 abundant gut bacteria, *Hafnia*, *Lactobacillus*, and *Enterobacter* were shared by most samples in the GG and GN groups, while *Pseudomonas*, *Arthrobacter*, and *Pantoea* were shared by most individuals of Bar-headed Geese.

To identify the gut microbiota compositions that showed significant differences between and within groups, we performed a comparative analysis using Linear discriminant analysis effect size (LEfSe). We set the LDA abundance threshold to 3. In the gut microbiota of Black-necked Cranes, *Vagococcus*, *Lactobacillus*, *Megamonas*, *Buttiauxella*, and *Hafnia* showed significant differences compared to the gut microbiota of Common Cranes and Bar-headed Geese. *Lysinibacillus*, *Paenibacillus*, *Lachnoclostridium*, *Enterobacter*, and *Klebsiella* were the classification units that showed significant differences in the gut of Common Cranes compared to the other two groups. *Methylobacterium*, *Agrobacterium*, *Rhizobium*, *Massilia*, *Helicobacter*, *Halomonas*, and *Pseudomonas* were the genera that showed significant differences between the gut microbiota of Bar-headed Geese and the other two groups (Figure 2H).

### 3.3. Analysis of dietary composition and diversity of the three species

**FIGURE 3:**
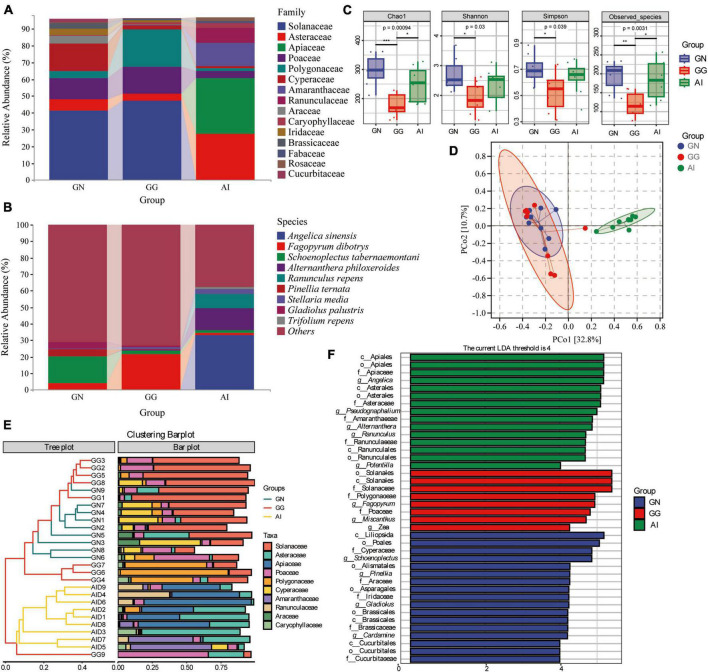
The composition of plant-based composition with relative abundance greater than 1% at the family **(A)** and species **(B)** levels. Alpha diversity in plant-based composition **(C)** of Black-necked Cranes, Common Cranes and Bar-headed Geese based on Chao1, Shannon, Simpson and Oberserved species indexes. PCoA analysis **(D)**, Cluster analysis **(E)** and LEfSe analysis **(F)** of plant-based composition in Black-necked Crane, Common Crane, and Bar-headed Goose.

The chloroplast *rbcL* gene was used to determine the plant-based composition of Black-necked Crane, Common Crane and Bar-headed Goose diets. At the family level, 9 families detected in group GN had relative abundances of more than 1%, including Solanaceae (41.41%), Asteraceae (6.60%), Poaceae (12.53%), Polygonaceae (4.09%), Cyperaceae (16.34%), Araceae (4.35%), Iridaceae (3.52%), Brassicaceae (3.43%) and Cucurbitaceae (2.10%). In group GG, 7 faimiles had a relative abundance of more than 1%, including Solanaceae (47.09%), Asteraceae (4.22%), Poaceae (16.14%), Polygonaceae (21.98%), Cyperaceae (2.29%), Caryophyllaceae (1.39%) and Fabaceae (1.07%) (Figure 3A). 10 of the top 15 faimiles in group AI had a relative abundance of more than 1%, including Asteraceae (27.28%), Apiaceae (33.43%), Poaceae (4.10%), Polygonaceae (1.65%), Cyperaceae (1.42%), Amaranthaceae (13.57%), Ranunculaceae (8.99%), Caryophyllaceae (2.67%), Fabaceae (1.58%) and Rosaceae (2.04%) (Figure 3A). At the species level, although the identification rate is low, we can still find some food species with a relative abundance greater than 1%. *Angelica sinensis* (33.15%), *Alternanthera philoxeroides* (13.26%), *Ranunculus repens* (8.86%), were only detected in group AI, the relative abundance of *Fagopyrum dibotrys* in group GN, GG and AI was 3.97, 21.71, and 1.52% seperately, the relative abundance of *Schoenoplectus tabernaemontani* in group GN, GG and AI was 16.22, 2.17, and 1.40% seperately, *Pinellia ternata* (4.32%) *and Gladiolus palustris* (3.52%) was only detected in group GN, *Stellaria media* was dected in both GG and AI, had the relative abundance 1.36 and 2.62% seperately. *Trifolium repens* (1.51%) was only dected in group AI (Figure 3B).

Alpha diversity measures were calculated for the plant-based components for a comparison among the three groups (Figure 3C). With respect to plant-based components, values for the four alpha diversity indexes were significantly higher in group GN than in group GG (*P < 0.05; **P < 0.01; ***P < 0.001), were higher in group GN than AI, but there were no significant difference. Values for all alpha diversity indexes were lower in group GG than in group AI, apart from the Shannon and Simpson index, the other two indexes had the significant difference. It is indicated that the food types and abundance of the Black-necked Crane and the Bar-headed Goose were both higher than those of the Common Crane.

To further analyze the plant-based components differences among groups, we used PCoA and ANOSIM (Figure 3D and Table 2). ANOSIM indicated that the difference among groups all showed *R* > 0, which means the difference between groups was greater than the difference within groups. The differences between group GN and GG were significant (*P* = 0.049 < 0.05), the differences between GN and AI, GG and AI were all significant (*P* = 0.001, Table 2).

**TABLE 2:** The ANOSIM analysis of OTUs based on Bray_Curtis between groups.

Group 1	Group 2	Sample size	R	*p*-value
All	–	27	0.636869	0.001
GN	GG	18	0.127572	0.049
GN	AI	18	0.929698	0.001
GG	AI	18	0.91941	0.001

To identify the similar plant-based dietary compositions between groups and within groups, we performed cluster analysis on the top 10 abundance-ranked plant-based food species at the family level and presented the results using an interactive bar chart (Figure 3E). It was found that there was a higher similarity between samples from the GN group and the GG group, with most of them clustering together. Except for GN9, the remaining eight individuals in the GN group clustered together, while the AI group and GG9 clustered separately. Solanaceae, Polygonaceae, and Cyperaceae were shared by most samples from the GG group and the GN group, while Asteraceae and Apiaceae were shared by most individuals of the Bar-headed Goose. Poaceae was shared by most individuals in the three groups. To identify the significantly different plant-based food compositions between groups and within groups, we conducted comparative analysis using LEfSe, with an LDA abundance threshold set at 4. In the GN group, Cyperaceae, Araceae, Iridaceae, Brassicales and Cucurbitaceae, Pinella, Gladiolus, and Cardamine were significantly different families and genera compared to the other two groups. Solanaceae, Polygonaceae, Poaceae, *Fagopyrum, Miscanthus, and Zea* were significantly different families and genera in the GG group compared to the other two groups. Apiaceae, Asteraceae, Amaranthaceae, Ranunculaceae, *Angelica, Pseudognaphalium, Alternanthera, Ranunculus*, and *Potentilla* were significantly different families and genera found in the Bar-headed Goose group compared to the other two groups (Figure 3F).

### 3.4. Spearman analysis between plant-based food components and gut microbiome and its functions

**FIGURE 4:**
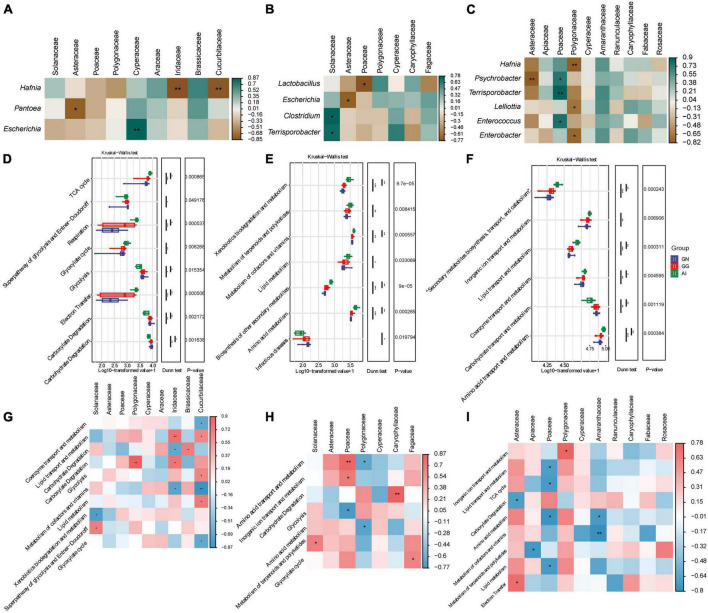
Correlation analysis between gut microbiota and plant-based food components of Black-necked Cranes **(A)**, Common Cranes **(B)** and Bar-headed Geese **(C)** seperately based on spearman algorithm. Analysis of significant differences in relative abundance of metabolism function and among GN, GG and AI based on the MetaCyc **(D)**, KEGG **(E)** and EggNOG **(F)** database. Correlation analysis between gut function and plant-based food components of Black-necked Cranes **(G)**, Common Cranes **(H)** and Bar-headed Geese **(I)** seperately based on spearman algorithm. *P < 0.05, **P < 0.01, and ***P < 0.001.

Spearman analysis were used to analyze the relationships between the relative abundance of taxa in the gut microbiota and plant-based food components of Black-necked Crane, Common Crane and Bar-headed Goose diets. In group GN (Figure 4A), Food with an abundance greater than 1% is only correlated with three bacteria with an abundance greater than 1%. The frequency of Iridaceae and Cucurbitaceae were significant negative correlation with *Hafnia;* the frequency of Asteraceae was significant negative correlation with *Pantoea*; only Cypetaceae was positively correlated with *Escherichia*. The other plant-based components were not significantly correlated with other gut microbial taxa. In group GG (Figure 4B), Solanaceae was significantly positively correlated with *Clostridium* and *Terrisporobacter*, while Asteraceae and Poaceae were negatively correlated with *Escherichia* and *Lactobacillus* respectively with significance. Polygonaceae, Cyperaceae, Caryophyllaceae and Fagaceae were correlated with *Lactobacillus*, *Escherichia*, *Clostridium* and *Terrisporobacter* with no significance. In group AI (Figure 4C), *Hafnia*, *Psychrobacter*, *Terrisporobacter*, *Lelliottia*, *Enterococcus* and *Enterobacter* had the correlation with Asteraceae, Apiaceae, Poaceae, Polygonaceae, Cyperaceae, Amaranthaceae, Ranunculaceae, Caryophyllaceae, Fabaceae and Rosaceae. Among them, Asteraceae was significantly negatively correlated with Psychrobacter; Polygonaceae was significantly negatively correlated with *Hafnia*, *Lelliottia* and *Enterobacter*. Only Poaceae was significantly positively correlated with *Psychrobacter*, *Terrispotobacter* and *Enterococcus*.

We performed functional enrichment analysis of secondary metabolic pathways using MetaCyc. Differential boxplot was used for result display, and we found a total of 8 significantly different secondary metabolic pathways, including Carbohydrate Degradation, Carboxylate Degradation, Electron Transfer, Glycolysis, Glyoxylate cycle, Respiration, Superpathway of glycolysis and Entner-Doudoroff, and TCA cycle (Figure 4D). Comparing to the AI group, the GN group showed significant enrichment in Carbohydrate Degradation, Carboxylate Degradation, Glycolysis, Superpathway of glycolysis and Entner-Doudoroff, while the AI group showed significant enrichment in the remaining four pathways. The enrichment levels of various functions in the GN group and GG group were approximately similar. This indicates that the metabolic pathways of red-crowned cranes and hooded cranes are similar, while they are significantly different from the metabolic pathways of bean geese. When analyzing using Kegg, we found 7 significantly different secondary pathways, including Infectious diseases, Amino acid metabolism, Biosynthesis of other secondary metabolites, Lipid metabolism, Metabolism of cofactors and vitamins, Metabolism of terpenoids and polyketides, and Xenobiotics biodegradation and metabolism. The enrichment of various functions in Black-necked Cranes and Common Cranes remained similar. There were significant differences in the enrichment levels of all seven secondary pathways between Black-necked Cranes and Bar-headed Geese, except for Infectious diseases. The bar-headed geese group showed higher enrichment levels in the other six pathways (Figure 4E), indicating that Bar-headed Geese had stronger synthesis and metabolic functions, implying that they may be healthier. The Common Cranes group showed intermediate enrichment levels, and the Black-necked Cranes group carried the highest load of pathogenic bacteria. When we performed secondary metabolic pathway enrichment analysis using the EggNOG databases, we found significant differences in 6 pathways, including Amino acid transport and metabolism, Carbohydrate transport and metabolism, Coenzyme transport and metabolism, Lipid transport and metabolism, Inorganic ion transport and metabolism, Secondary metabolites biosynthesis, transport, and catabolism (Figure 4F). Except for Carbohydrate transport and metabolism, the Bar-headed Geese group showed significant enrichment in the other five metabolic pathways.

Spearman analysis was also used to analyze the relationships between the relative abundance of taxa in the gut microbiota functions and plant-based food components of the Black-necked Crane, Common Crane, and Bar-headed Goose diets. In the GN group (Figure 4G), Food components with abundance greater than 1% were found to be correlated with 8 families at the Order level, including Lipid transport and metabolism, Glycolysis, Carbohydrate degradation, and 10 other secondary metabolic pathways. In GG group (Figure 4H), food components with abundance greater than 1% were found to be correlated with 7 families at the Order level, including Amino acid transport and metabolism, Carbohydrate degradation, Amino acid metabolism, and 7 other secondary metabolic pathways. In group AI (Figure 4I), food components with abundance greater than 1% were found to be correlated with 10 families at the Order level, including Inorganic ion transport and metabolism, Lipid transport and metabolism, TCA cycle, and 9 other secondary metabolic pathways.

## 4. Discussion

### 4.1. The gut microbiota and the roles of three birds

This study selected the Black-necked Crane and the Common Crane, which are similar in body size, feeding type, and habitat, and the smaller-sized Bar-headed Goose with a high ecological niche overlap as the research objects to explore the differences in gut microbiota composition and function among co-distributed species and hope to identify possible reasons. In our study, the main phyla of gut microbiota in Black-necked Cranes were Proteobacteria (69.26%) and Firmicutes (29.51%); the main phyla of gut microbiota in Common Cranes were Proteobacteria (63.61%), Firmicutes (32.40%), and Actinobacteria (2.99%); the main phyla of gut microbiota in Bar-headed Geese were Proteobacteria (63.61%), Firmicutes (32.40%), Actinobacteria (2.99%), and Bacteroidetes (3.42%). This research result is different from the dominant phyla of most birds: Proteobacteria, Firmicutes, Actinobacteria, and Bacteroidetes (Hird, 2017; Wang et al., 2020; Maraci et al., 2021). Proteobacteria is a phylum with highly complex physiological functions. Some members can degrade cellulose, which can help the host effectively utilize carbon sources and accumulate energy, but abnormal proliferation of Proteobacteria may be related to metabolic disorders of gut microbiota and malnutrition of hosts (Shin et al., 2015). Firmicutes play an important role in maintaining intestinal stability and assisting digestion. Several previous studies have shown that many members of Firmicutes and Bacteroidetes can express carbohydrate-active enzymes to help hosts hydrolyze and utilize carbohydrates (El Kaoutari et al., 2013). Compared with Firmicutes, Bacteroidetes contains more glycan hydrolases, which play an important role in polysaccharide fermentation and can improve the utilization rate of nutrients by hosts during polysaccharide decomposition process (Bäckhed et al., 2004; El Kaoutari et al., 2013; Yang et al., 2017). Bacteroidetes and Proteobacteria are usually considered to be related to the intake of Poaceae (Dong et al., 2019). The content of Actinobacteria will increase with the increase of host’s intake of cellulose (Lee et al., 2015).

At the species level, *Hafnia*, which is shared by most GG and GN group samples, is an opportunistic pathogen that can cause infections in the human intestinal flora (Janda and Abbott, 2006). *Hafnia alvei* is an opportunistic pathogen associated with human intestinal and extraintestinal infections (Song et al., 2017). *Lactobacillus* can effectively inhibit the occurrence of diseases (Shi et al., 2020), and *Lactobacillus salivarius* can be added to chicken feed as a probiotic to improve chicken production performance and overall intestinal health (Sureshkumar et al., 2021; Xu et al., 2022). *Enterobacter* can also be used as a probiotic to promote host growth (Augustinos et al., 2015). These three bacteria, as the main components of the intestinal microbiota of Black-necked Cranes and Common Cranes, are considered to be adaptive mechanisms for achieving a balance of gut microbiota with the body.

*Pseudomonas* and *Arthrobacter* are shared by most Bar-headed Goose individuals. *Pseudomonas* and *P. graminis* have the function of amylases, while *Arthrobacter* can decompose both Cellulolytic and xylanolytic (Briones-Roblero et al., 2017), indicating that these bacteria shared by multiple Bar-headed Goose individuals are involved in their metabolism. In addition, through Lefse analysis, we found that in addition to *Hafnia* (41.41%) and *Lactobacillus* (21.75%), *Vagococcus* (1.48%) is a significant difference in the gut microbiota of Black-necked Cranes compared to Common Cranes and Bar-headed Geese, and this bacterium is also a probiotic (Fregeneda-Grandes et al., 2023). In addition to *Enterobacter* (10.69%), *Lysinibacillus* (1.72%), *Paenibacillus* (1.21%), and *Klebsiella* (4.34%) are significantly different operational taxonomic units in the Common Crane’s intestines compared to the other two groups. *Lysinibacillusa* was also a promising probiotic candidate for intestinal health (Chen et al., 2023). *Paenibacillus* and *Klebsiella* isolates play a role as pathogens (Sáez-Nieto et al., 2017). The genus that is significantly different from the other two groups in the Bar-headed Goose’s intestinal microbiota is *Pseudomonas* (30.04%). Therefore, the bacteria with higher proportions in Black-necked Cranes and Common Cranes are either probiotics or pathogens, while the bacteria with higher proportions in Bar-headed Geese are related to their metabolism of food.

### 4.2. The feeding strategies, nutritional components, and functional analysis of food for three sympatric species

The feeding strategies, nutritional components, and functional analysis of food for three sympatric species are as follows. Since the species were not fully identified at the species level, we chose to analyze the families and successfully identified species. More than 80% of the food of the Black-necked Crane is composed of three families: Solanaceae (41.41%), Cyperaceae (16.34%) with *S. tabernaemontani* (16.22%), and Poaceae (12.53%). More than 80% of the food of the Common Crane is composed of Solanaceae (47.09%), Polygonaceae (21.98%) with *F. dibotrys* (21.71%), and Poaceae (16.14%). These three families happen to be the biomarkers that show significant differences between the Common Crane and the other two groups in Lefse analysis. More than 80% of the food of the Bar-headed Goose is composed of Asteraceae (27.28%), Apiaceae (33.43%) with *A. sinensis* (33.15%), Amaranthaceae (13.57%) with *A. philoxeroides* (13.26%), and Ranunculaceae (8.99%) with *R. repens* (8.86%). These three families are also biomarkers that show significant differences between the Bar-headed Goose and the other two groups in Lefse analysis.

Although the species contained in Solanaceae were not successfully identified, through actual investigation, we know that Black-necked Cranes and Common Cranes mainly feed on potatoes as their main food during the wintering period. Carbohydrates are the dominant nutrient of potatoes (Górska-Warsewicz et al., 2021). Potatoes and potato components have favorable impacts on several measures of cardiometabolic health in animals, including improving lipid profiles, and decreasing markers of inflammation (McGill et al., 2013). *S. tabernaemontani* contained five *Macrocyclic glycosides* and has a positive effect on diuretic, edema, and urine impassable (Peng et al., 2021). Combined with field observations and reports from others, plants in the Poaceae family are mainly corn. Corn kernels contain starch (61% to 78%), non-starch polysaccharides, protein, and lipids. They promote postprandial glycemic/insulinemic responses, lipid metabolism, colon health, and mineral absorption (Ai and Jane, 2016). *F. dibotrys* is one of the long-history used traditional medicine in China. Its rhizomes have significant anti-inflammatory, antibacterial, and anticancer activities (Zhang et al., 2023). Among the main diets of Bar-headed Geese, the Asteraceae family is the only one that we cannot determine its specific species, which is mainly composed of vegetables, medicinal plants, and ornamental plants. *A.sinensis* is commonly used as a traditional medicinal herb or a food/dietary supplement in Asia, Europe, and North America, has the effects of tonifying blood and activating blood circulation (Ping et al., 2023). *A. philoxeroides* is an invasive alien plant with antibacterial and antiviral functions (Rattanathongkom et al., 2009; Peng et al., 2021). *R. repens* have compounds that showed potent inhibitory activity against urease. Urease is detrimental to human and animal health (Khan et al., 2006). Therefore, more than 50% of the food for Black-necked Cranes and Common Cranes is similar, consisting of potatoes and corn. To meet the dietary needs of wintering cranes in Cao Hai, these two foods are only planted but not harvested locally, so these two foods are relatively abundant in Cao Hai wetlands. For more than 40% of other foods, there are significant differences between Black-necked Cranes and Common Cranes; therefore there is no competition for food between wintering Black-necked Cranes and Common Cranes in Cao Hai. The Bar-headed Goose coexisting with these two cranes has significant differences from them in terms of food types; therefore this is why they can coexist harmoniously despite foraging sympatrically.

### 4.3. Effects of host genetic background and diet on gut microbiome structure and functions

Alpha diversity analysis showed no significant differences between the gut microbiota of Black-necked Cranes, Common Cranes, and Bar-headed Geese. Beta diversity combined with ANOSIM analysis found no significant differences between Black-necked Cranes and Common Cranes, but there were significant differences between Black-necked Cranes and Bar-headed Geese, as well as between Common Cranes and Bar-headed Geese. Clustering analysis and correlation network analysis of gut microbiota showed that Black-necked Cranes and Common Cranes have similar gut microbiota composition, while the gut microbiota of Bar-headed Geese is very different. Studies have shown that the structure of vertebrate gut microbiota changes with the seasons, mainly due to the adaptive changes in the gut microbiota of different species due to different dietary structures at different times (You et al., 2022). However, in this study, alpha diversity analysis showed that the abundance and diversity of food types within the Black-necked Crane group were significantly higher than those of gray cranes and higher than those of Bar-headed Geese but not significantly different. The diversity of food types within the gray crane group was lower than that of Bar-headed Geese, but not significantly different, and the richness of food was significantly lower than that of Bar-headed Geese. This result is consistent with the clustering analysis of plant-based foods for the three bird species. PcoA analysis revealed significant differences in food intake between Black-necked Cranes, gray cranes, and Bar-headed Geese. Black-necked Cranes and gray cranes, which have significant differences in food intake, do not have significant differences in their gut microbiota. The reason may be that compared to Bar-headed Geese, Black-necked Cranes and gray cranes have a closer phylogenetic relationship, which is the main reason why they have similar microbiota. The alpha diversity of Bar-headed Goose gut microbiota is the highest, while its food alpha diversity is the lowest. Spearman analysis also showed that there are not many species that are correlated with food and gut bacteria. Corn straw powder can increase the abundance of Proteobacteria and Actinobacteria, while adding alfalfa can increase the abundance of Firmicutes and Bacteroidetes (Jia et al., 2022). However, Black-necked Cranes and gray cranes that eat more corn did not show a high proportion of Actinobacteria. Bar-headed Geese that eat more cellulose did not show a high proportion of Firmicutes. Therefore, we support the research conclusion that the host genetic background rather than food is the dominant factor determining the gut microbial community (Li et al., 2021; Lu et al., 2022).

It is generally believed that the higher the alpha diversity of gut microbiota, the higher its ability to absorb nutrients and resist pathogens (Le Chatelier et al., 2013; Ganz et al., 2017). In this study, Kegg functional analysis showed that Black-necked Cranes are more likely to be infected with diseases, which may be related to Hafnia alvei infection in their gut microbiota. Bar-headed Geese are least likely to be infected with diseases, possibly because their main foods A. sinensis, A. philoxeroides and R. repens all have certain health functions, enhancing their resistance to disease and promoting their health. Dietary regulation can affect the metabolic or immune function of gut microbiota, thereby indirectly or directly participating in physiological metabolism, digestive function and immune system (Rinninella et al., 2019; Guo et al., 2021). Amino acid metabolism, biosynthesis of other secondary metabolites, lipid metabolism, metabolism of cofactors and vitamins, metabolism of terpenoids and polyketides, xenobiotics biodegradation and metabolism related to nutrition and health are all enriched to the highest degree in Bar-headed Goose gut microbiota function and are significantly different from Black-necked Cranes and gray cranes. This was also confirmed again in the Egg-Nog database. Gray cranes are less likely to be infected with diseases than Black-necked Cranes, possibly due to their large intake of buckwheat. Spearman analysis showed that food is almost correlated with all secondary metabolites. Black-necked Cranes and gray cranes eat high-starch foods such as corn and potatoes with a proportion of more than 50% in their food, while most of Bar-headed Goose’s food is composed of cellulose. The functional prediction of gut microbiota for three bird species shows that Black-necked Cranes and gray cranes mainly supply energy through degradation of carbohydrates, degradation of carboxylates, sugar fermentation, while Bar-headed Geese supply energy through acetaldehyde cycle, respiration and TCA cycle for their bodies. This also directly shows that the different energy production methods of three bird species are related to their food composition. Therefore, we believe that the function of bird gut microbiota is related to food, but its composition is mainly affected by the host itself.

## 5. Conclusion

We chose Black-necked Cranes, Common Cranes, and Bar-headed Geese that are distributed in the same domain as the research objects in order to eliminate the impact of the natural environment on their gut microbiota. Under the relatively accurate analysis of food composition, we explored the impact of hosts and diet on bird gut microbiota. It was found that Black-necked Cranes and Common Cranes, which are closely related, have significant differences in diet but have similar microbial structure and function. Bar-headed Geese, which are more distantly related to these two cranes, have significant differences in diet, gut microbiota composition and function. This shows that the difference in food intake is the main reason for the coexistence of three bird species in the same domain. The host genetic background is the dominant factor determining the composition of gut microbiota, and food dominates the differentiation of metabolic functions of gut microbiota in three bird species, the consumption of medicinal plants promotes the gut health of birds. In addition, it is recommended that the competent authorities monitor local corn, potatoes, *F. dibotrys*, *S. tabernaemontani*, *A. philoxeroides*, *R. repens* and other major foods for three bird species during their wintering period, and fully consider these bird species’ preferred foods when restoring wetlands or providing artificial food supplies, especially medicinal plants.

## Data availability statement

The datasets presented in this study can be found in online repositories. The names of the repository/repositories and accession number(s) can be found below: https://www.ncbi.nlm.nih.gov/genbank/, PRJNA992803.

## Ethics statement

This research approved by the Research Ethics Committee of Guizhou Normal University (Approval No. GZNUECEE-2022-009). We only collected feces for relevant studies, did not involve capture or any direct manipulation or disturbance of wild birds in the fieldwork.

## Author contributions

YW: Conceptualization, Formal analysis, Writing – original draft, Writing – review and editing. HS: Conceptualization, Formal analysis, Writing – original draft, Writing – review and editing. ZL: Investigation, Writing – review and editing. YZ: Investigation, Writing – review and editing. XL: Investigation, Writing – review and editing. XZ: Investigation, Writing – review and editing.

## Footnotes

1
https://www.genescloud.cn

2
https://www.kegg.jp/

3
https://metacyc.org/
