# Supplementary material for: Host genetic background rather than diet-induced gut microbiota shifts of sympatric black-necked crane, common crane and bar-headed goose
Source: Front Microbiol. 2023 Oct 12;14:1270716. doi: 10.3389/fmicb.2023.1270716 (PMC10625752; doi:10.3389/fmicb.2023.1270716)
Supplement: Supplementary file 1 [file Table_1.DOCX]

Table S1 Results of OTU processing of Illumina Mi Seq amplification products of *rbcl* gene

| Sample  Step | Input | Merged | Filtered | Non chimeric | Non singleton |
| --- | --- | --- | --- | --- | --- |
| AID1 | 107422 | 106225 | 104869 | 98206 | 98203 |
| AID2 | 116246 | 114805 | 113282 | 100205 | 100197 |
| AID3 | 74706 | 74283 | 73336 | 67313 | 67309 |
| AID4 | 96414 | 95819 | 94747 | 84670 | 84664 |
| AID5 | 101819 | 101091 | 99484 | 78713 | 78691 |
| AID6 | 87810 | 86850 | 85568 | 74569 | 74562 |
| AID7 | 95947 | 95437 | 94088 | 76861 | 76856 |
| AID8 | 102465 | 101789 | 100352 | 86831 | 86822 |
| AID9 | 99764 | 99175 | 97898 | 79208 | 79192 |
| GN1 | 134025 | 123437 | 121628 | 106902 | 106893 |
| GN2 | 147185 | 140947 | 138566 | 119078 | 119064 |
| GN3 | 135909 | 127800 | 125687 | 113129 | 113118 |
| GN4 | 135213 | 129869 | 127725 | 111027 | 111006 |
| GN5 | 138754 | 127607 | 125631 | 112196 | 112184 |
| GN6 | 143694 | 130106 | 128096 | 114998 | 114994 |
| GN7 | 141254 | 127252 | 125352 | 108660 | 108642 |
| GN8 | 138559 | 128150 | 125999 | 113984 | 113969 |
| GN9 | 133419 | 124892 | 123053 | 114599 | 114577 |
| GG1 | 131606 | 114128 | 112397 | 106309 | 106303 |
| GG2 | 135086 | 127888 | 125772 | 115600 | 115592 |
| GG3 | 136644 | 123498 | 121528 | 105495 | 105486 |
| GG4 | 139903 | 134608 | 132411 | 117092 | 117083 |
| GG5 | 134646 | 125660 | 123795 | 115718 | 115716 |
| GG6 | 143685 | 137867 | 135691 | 130272 | 130268 |
| GG7 | 147110 | 141928 | 139773 | 132240 | 132237 |
| GG8 | 146632 | 144201 | 141924 | 130361 | 130357 |
| GG9 | 134257 | 130291 | 128324 | 115907 | 115905 |
