# Supplementary material for: Host genetic background rather than diet-induced gut microbiota shifts of sympatric black-necked crane, common crane and bar-headed goose
Source: Front Microbiol. 2023 Oct 12;14:1270716. doi: 10.3389/fmicb.2023.1270716 (PMC10625752; doi:10.3389/fmicb.2023.1270716)
Supplement: Supplementary file 2 [file Table_2.DOCX]

Table S2 ASV processing results of Illumina Mi Seq amplification products of gut bacteria

| Sample ID | Input | Filtered | Denoised | Merged | Non-chimeric | Non-singleton |
| --- | --- | --- | --- | --- | --- | --- |
| AID1 | 106456 | 93948 | 92528 | 90671 | 79967 | 79586 |
| AID2 | 80660 | 72282 | 71217 | 70326 | 59191 | 59032 |
| AID3 | 95743 | 86623 | 85116 | 82691 | 70672 | 70127 |
| AID4 | 104909 | 93597 | 92827 | 91850 | 85928 | 85812 |
| AID5 | 101157 | 90105 | 88390 | 86177 | 60944 | 60387 |
| AID6 | 114057 | 102994 | 100365 | 96157 | 78773 | 78049 |
| AID7 | 122439 | 105474 | 102970 | 98294 | 78958 | 78167 |
| AID8 | 90000 | 80050 | 79488 | 78740 | 53089 | 52983 |
| AID9 | 130165 | 116147 | 114249 | 110648 | 98727 | 98229 |
| GN1 | 109049 | 100742 | 99969 | 98957 | 88536 | 88330 |
| GN2 | 101389 | 93478 | 92809 | 91894 | 80144 | 80018 |
| GN3 | 100635 | 92714 | 91598 | 89439 | 70135 | 69571 |
| GN4 | 109341 | 100527 | 99967 | 99233 | 78984 | 78882 |
| GN5 | 106814 | 97931 | 96735 | 94800 | 73501 | 73199 |
| GN6 | 104352 | 95900 | 94761 | 93290 | 78749 | 78286 |
| GN7 | 109314 | 101660 | 100913 | 100010 | 76305 | 76159 |
| GN8 | 108686 | 100686 | 100158 | 99491 | 96105 | 96021 |
| GN9 | 110562 | 101952 | 101192 | 100209 | 70303 | 70106 |
| GG1 | 105702 | 98206 | 95326 | 89589 | 57443 | 56318 |
| GG2 | 99715 | 92059 | 91356 | 90658 | 85907 | 85810 |
| GG3 | 100759 | 93075 | 91734 | 89628 | 71940 | 71269 |
| GG4 | 106948 | 99706 | 99036 | 98085 | 83073 | 82923 |
| GG5 | 107110 | 99359 | 98700 | 97510 | 81361 | 81217 |
| GG6 | 108626 | 101149 | 100686 | 99795 | 88380 | 88307 |
| GG7 | 106007 | 98029 | 97395 | 96759 | 87474 | 87379 |
| GG8 | 100903 | 91219 | 88862 | 84258 | 54190 | 52654 |
| GG9 | 102862 | 96823 | 96284 | 95759 | 78215 | 78105 |
| Total | 2844360 | 2596435 | 2564631 | 2514918 | 2066994 | 2056926 |
