# Supplementary figures and images for: Host genetic background rather than diet-induced gut microbiota shifts of sympatric black-necked crane, common crane and bar-headed goose
Source: Front Microbiol. 2023 Oct 12;14:1270716. doi: 10.3389/fmicb.2023.1270716 (PMC10625752; doi:10.3389/fmicb.2023.1270716)

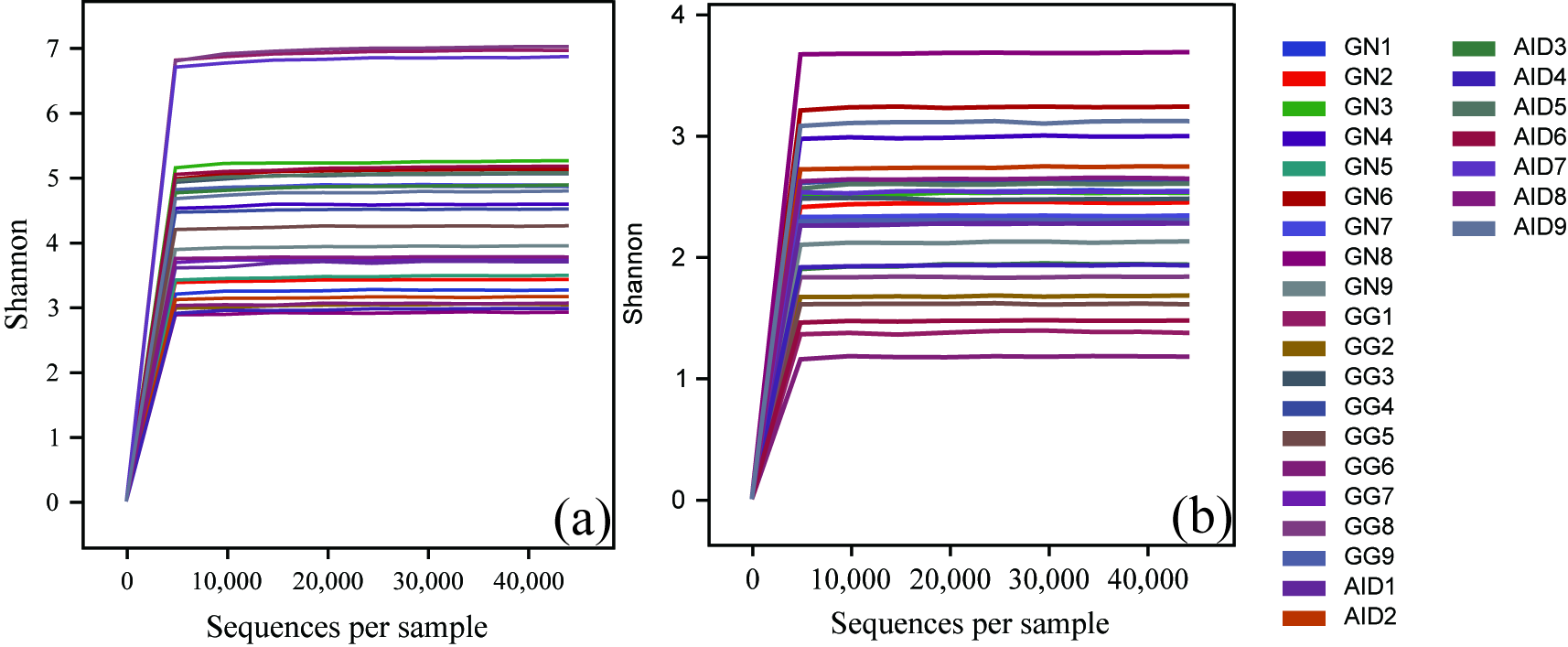

Supplement: Supplementary file 3 [file Image_1.TIF]
